# Fox-Fordyce Disease in Women: A Case Report Highlighting Laser-Based Interventions

**DOI:** 10.7759/cureus.85490

**Published:** 2025-06-06

**Authors:** Saud S Al Mohrij, Yasser A Ghobara, Ahmed Al-Issa

**Affiliations:** 1 College of Medicine, Almaarefa University, Riyadh, SAU; 2 Dermatology, Derma Clinic, Riyadh, SAU

**Keywords:** apocrine glands, co₂ laser, dermatology, dermatology case report, erbium:yag laser, fox–fordyce disease

## Abstract

Fox-Fordyce disease (FFD) is a rare, chronic skin disorder affecting apocrine sweat glands, predominantly in women. It manifests as pruritic, dome-shaped papules in areas such as the axillae and pubic region. Treatment remains challenging, with limited success from conventional therapies.

We report two cases of Saudi women aged 27 and 28 years presenting with hyperpigmented axillary papules and mild pruritus, consistent with FFD. One patient had no comorbidities, while the other had a known history of sickle cell anemia. Both cases were managed with laser therapy (erbium:yttrium aluminum garnet (YAG) in case 1 and ablative CO₂ in case 2) using precise protocols and follow-up plans. Clinical images demonstrated visual improvement post treatment. Adjunctive topical therapy was administered to manage inflammation and prevent hyperpigmentation.

The cases underscore the typical presentation of FFD and the potential efficacy of laser-based interventions, even in patients with complex medical histories. Notably, the presence of sickle cell anemia did not exacerbate FFD manifestations, supporting the current treatment modalities. While hormonal therapy was not employed, its relevance remains a subject for future exploration.

Laser therapy offers a promising approach for managing refractory FFD. Further studies are warranted to evaluate long-term outcomes and explore hormone-modulating strategies tailored to FFD’s pathophysiology.

## Introduction

Fox-Fordyce disease (FFD) is an uncommon chronic skin condition that primarily affects apocrine sweat glands, resulting in itchy papules in areas such as the axillae, areolae, and pubic region [1]. While the precise cause of FFD is not well understood, it is believed to involve the blockage of apocrine sweat gland ducts, which can lead to duct rupture and subsequent inflammation [2]. Hormonal factors, especially those related to estrogen, are also considered significant, as the condition mainly affects women and tends to worsen during hormonal changes such as puberty, pregnancy, and menstruation [3]. Managing FFD poses therapeutic challenges due to its chronic nature and the limited success of existing treatments. Topical treatments like corticosteroids, retinoids, and clindamycin may offer some relief but often do not result in complete resolution [4]. Systemic therapies, including hormonal treatments such as oral contraceptives or anti-androgens, have been investigated due to the suspected hormonal involvement in FFD, but their effectiveness can be inconsistent, and they may have significant side effects [5]. Additionally, surgical options like excision or laser therapy have been used in cases that do not respond to other treatments, but these methods carry the risks of scarring and recurrence [6]. The persistent and often resistant nature of FFD highlights the necessity for more effective and targeted treatment options.

## Case presentation

Study design

This is a retrospective case study conducted at Derma Clinic, Riyadh, Saudi Arabia. The patients presented in March 2018 (case 1) and November 2020 (case 2) to the dermatology outpatient clinic. The two cases were not consecutive; they were included based on the availability of complete clinical documentation and photographic follow-up before and after treatment.

Case 1

A 27-year-old Saudi woman presented to the clinic with concerns about hyperpigmentation and the appearance of multiple papules in both axillary regions. The patient reported mild, intermittent itching that caused minimal discomfort. On clinical examination, numerous round, dome-shaped papules were observed, primarily distributed across various areas of the axilla. The papules exhibited a slightly dark reddish hue.

Her vital signs were stable, and there were no systemic symptoms present. The patient denied any significant past medical history, including systemic diseases, recent infections, or previous occurrences of similar skin lesions. Additionally, she was not on any medications at the time of presentation.

As part of her treatment, the patient underwent a session of erbium:yttrium aluminum garnet (YAG) laser: 2940 nm laser therapy targeting the affected areas. The procedure was performed using the ACTION II™ device (Lutronic, Goyang-si, South Korea) with the following parameters: 1 mm spot size, 50 j/cm² fluence, and 5 Hz frequency. Topical lidocaine 5% cream was applied 45 minutes before the procedure. She had no prior history of adverse skin reactions to laser therapies.

**Figure 1:**
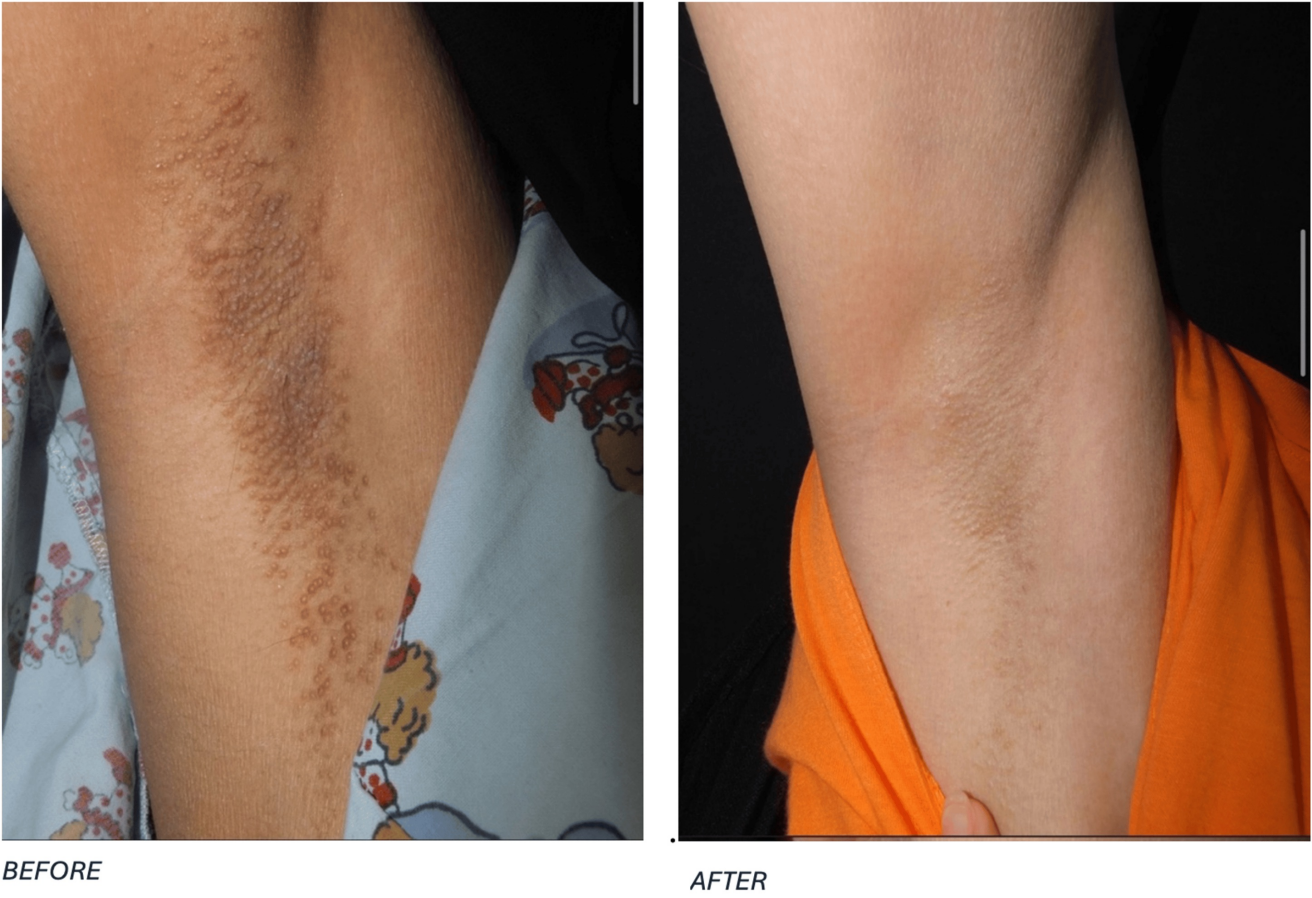
The condition of the right axilla in case 1, showcasing the patient’s skin before and after undergoing treatment.

**Figure 2:**
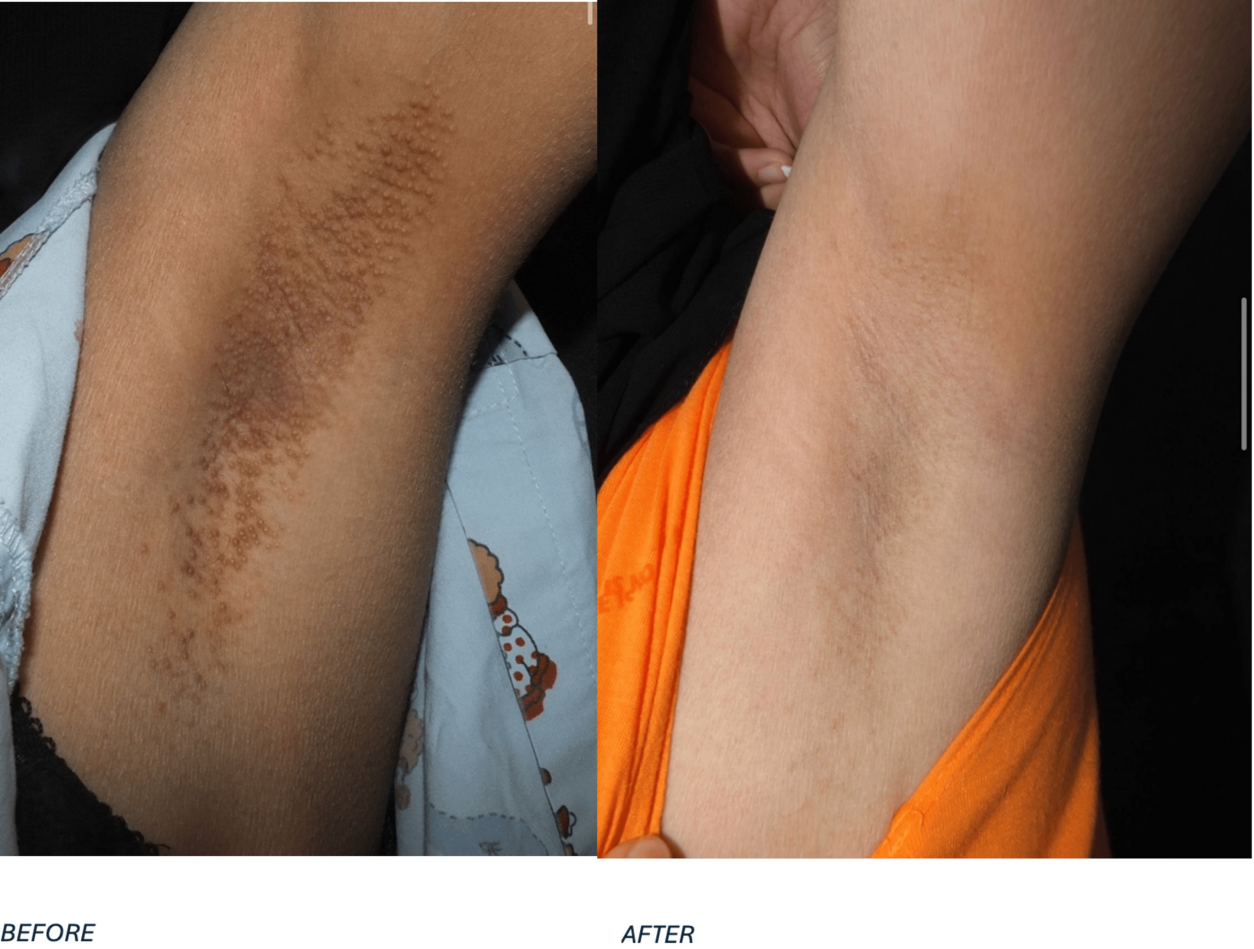
The condition of the left axilla in case 1, presenting a comparison of the skin before and after treatment.

Following the treatment, the patient was advised to use mometasone furoate cream twice daily for one week as part of her home treatment regimen. Further follow-up evaluations were performed on April 13, 2018, and November 15, 2020. At the final follow-up, there was no evidence of lesion recurrence. The clinical progression of the patient in case 1 is visually documented in Figure 1, which displays the condition of the right axilla before and after laser treatment. Similarly, Figure 2 illustrates the left axilla, showing a comparative view of the skin lesions pre- and post intervention.

Case 2

A 28-year-old Saudi woman with a known history of sickle cell anemia (SCA) presented to the clinic with mild, intermittent itching, describing it as low in intensity and only mildly bothersome, with hyperpigmentation and the appearance of papules in both axillary regions. On clinical examination, multiple round, dome-shaped papules were observed, slightly dark reddish in color, and distributed throughout the axillary areas.

Her vital signs were stable, and there were no associated systemic symptoms. Her medical history was notable for SCA, for which she was being treated with vitamin D and hydroxyurea. Aside from her SCA, she had no recent infections or other underlying systemic diseases and had not experienced similar eruptions in the past. Furthermore, there was no history of adverse reactions following previous laser treatments.

To address her condition, the patient underwent a session of ablative CO2 laser therapy using the eCO2™ device (Lutronic). The procedure was performed with the following settings: 50 mJ energy, 50 Hz pulse rate, point scan type, 30 watts power, and dynamic mode. Prior to the laser treatment, 3 ml of lidocaine 1% was injected for local anesthesia. Following the procedure, clobetasol propionate 0.05% ointment was topically applied to the treated areas, and the patient was advised to use mometasone furoate cream twice daily for one week as part of her home treatment regimen. Subsequent follow-up visits occurred on November 24, 2020, January 26, 2021, and March 28, 2021, to monitor healing and assess treatment efficacy.

**Figure 3:**
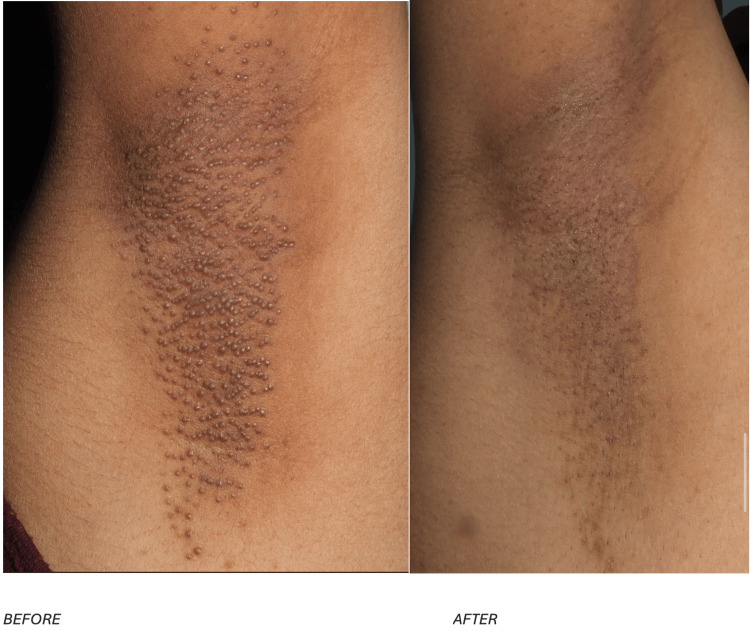
The condition of the right axilla in case 2, displaying a comparison of the skin before and after treatment.

**Figure 4:**
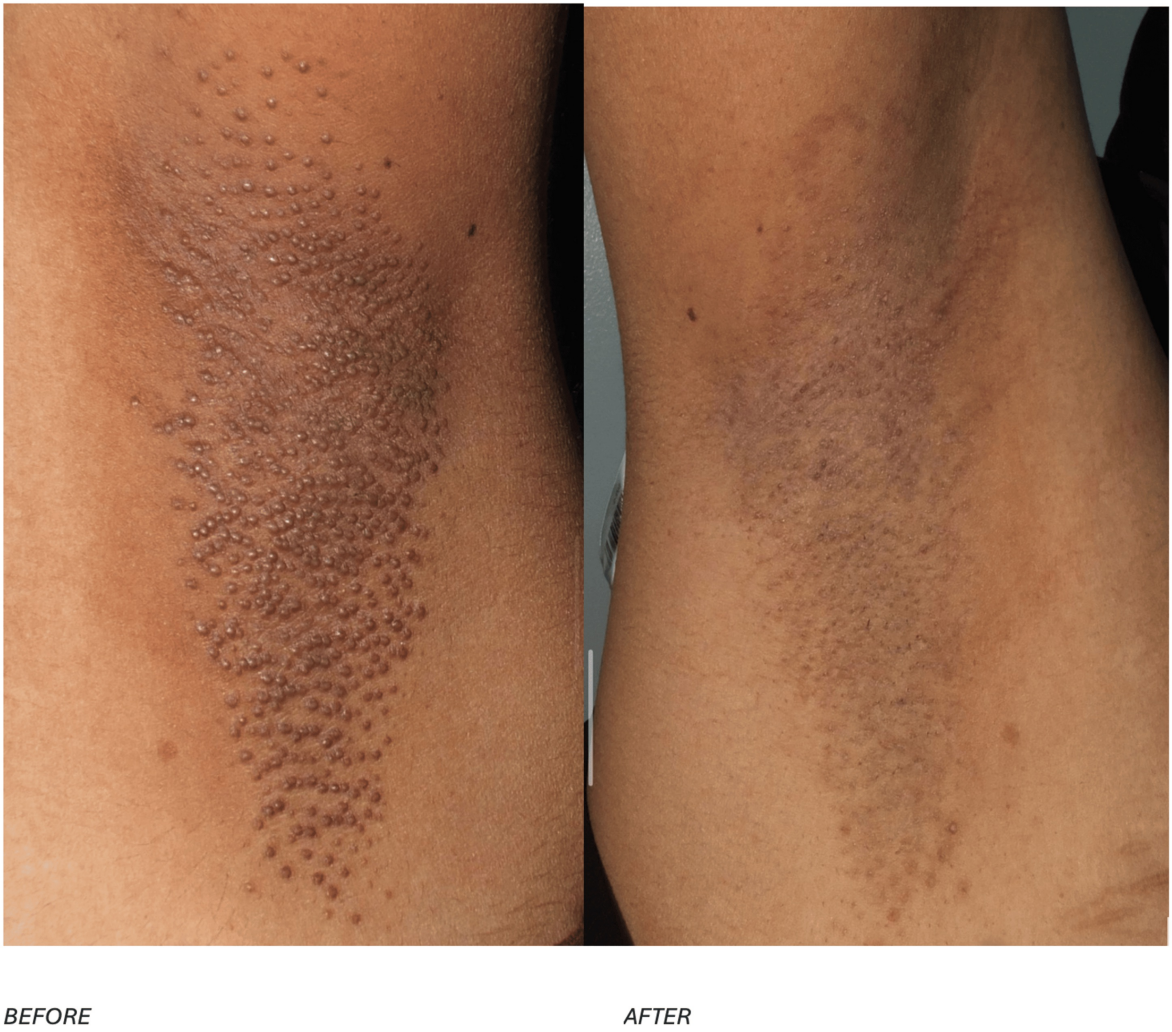
The condition of the left axilla in case 2, highlighting the skin's appearance before and after the treatment.

To complement the clinical description, Figure 3 presents the appearance of the right axilla before and after treatment, while Figure 4 demonstrates the corresponding changes in the left axilla.

Both patients were clinically followed up after their respective laser treatments. During these visits, no recurrence of papules or pruritus was observed, and both patients reported satisfaction with the cosmetic and symptomatic outcomes. While these short-term results are encouraging, given the chronic and relapsing nature of Fox-Fordyce disease, longer-term follow-up is essential to evaluate sustained treatment efficacy and detect potential recurrence.

## Discussion

The two cases presented in this report exhibit the classic clinical features of FFD and demonstrate that these features remain consistent even in patients with complex comorbidities. Both individuals were young women who developed multiple dome-shaped, follicular papules in the axillary regions, accompanied by intermittent pruritus - a presentation characteristic of FFD [1]. In the second case, the patient’s concurrent SCA and its treatment with hydroxyurea did not appear to influence FFD. (Hydroxyurea can cause cutaneous side effects such as hyperpigmentation and ulcerations, but it has not been linked to FFD.) Notably, the severity of FFD in the patient with SCA was similar to that of the patient without any systemic illness, suggesting that neither SCA nor its treatment exacerbated the condition. This observation is clinically important because it indicates that standard FFD management can be effectively applied even in patients with significant comorbid conditions.

Both patients in our series underwent laser therapy, reflecting an aggressive approach suitable for refractory FFD [1]. In case 1, an erbium:YAG laser was used to ablate the affected areas. This laser targets superficial skin layers and was employed to destroy the obstructed follicular apocrine units, causing the eruption. Notably, Han et al. [5] reported a similar approach in a case of areolar FFD, where a fractional 1550-nm erbium-glass laser (combined with surgical excision) successfully led to lesion clearance. Our case 1 patient likewise experienced a marked reduction in axillary papules following erbium laser treatment, consistent with the improvement noted by Han et al. [5]. In case 2, we utilized an ablative CO2 laser to treat the axillary lesions. Ablative CO2 lasers effectively diminish FFD papules by destroying the affected apocrine sweat glands [1]. The patient in case 2 showed significant improvement in lesion size and pruritus after this treatment.

Despite these favorable responses, one must consider the limitations and risks of laser therapy in FFD. Ablative laser procedures can result in scarring, post-inflammatory hyperpigmentation, and eventual recurrence of lesions [1]. In our cases, a topical corticosteroid was applied during the recovery period to help minimize post-laser inflammation and pigmentary change. It is also noteworthy that, in rare instances, laser treatments themselves have been implicated in precipitating FFD. Zargari and Azimi [6] reported a case in which a patient developed axillary FFD as an adverse effect of cosmetic diode-laser hair removal, presumably due to laser-induced damage to the follicular infundibulum leading to apocrine duct occlusion. This cautionary observation [6] underscores the importance of careful patient selection and counseling when considering laser therapy for FFD, as well as the need for diligent long-term follow-up given the chronic, relapsing nature of the condition [1].

Aside from laser ablation, other therapeutic modalities have been explored in FFD. Given the female predominance of FFD, hormonal manipulation is one considered avenue of treatment. Thiboutot and Chen [2] have discussed how anti-estrogenic or anti-androgen therapies (such as estrogen-progestin oral contraceptives or spironolactone) can benefit hormone-responsive dermatoses. In FFD, a few cases have attempted hormonal therapy with sporadic success [1]; however, robust evidence is lacking, and these treatments carry potential side effects [2]. Neither of our patients underwent hormonal intervention, but this option could be contemplated in select cases where endocrine factors are suspected to contribute to disease activity. In addition to hormonal approaches, medical (non-procedural) therapies have shown efficacy in some reports. George et al. [3] described two patients who experienced significant improvement of FFD with twice-daily topical clindamycin, demonstrating that a less invasive, anti-inflammatory approach can achieve symptom control in certain cases. Similarly, Effendy et al. [4] reported a male patient who responded favorably to oral isotretinoin, highlighting the potential of retinoid therapy to reduce follicular plugging and inflammation in FFD. These cases [3,4] illustrate that conservative treatments can sometimes manage FFD effectively. Nevertheless, in many patients, including those in our report, such measures prove inadequate, necessitating more aggressive interventions like laser therapy. Ultimately, the management of FFD should be individualized, and further research is needed to identify consistently effective treatments for this challenging chronic condition [1].

## Conclusions

FFD remains a challenging dermatological condition to manage due to its chronic nature and the limited efficacy of current treatments. The cases presented in this report underscore the typical clinical features of FFD and highlight the ongoing need for more effective and targeted therapeutic options. Moving forward, future research should prioritize the development of novel therapeutic strategies that target the underlying pathophysiology of FFD, with the goal of improving both symptom control and long-term patient outcomes.
